# Impact of Paired Remote Ischemic Preconditioning on Postreperfusion Syndrome in Living-Donor Liver Transplantation: A Propensity-Score Matching Analysis

**DOI:** 10.3390/medicina60111830

**Published:** 2024-11-07

**Authors:** Jaewon Huh, Min Suk Chae

**Affiliations:** Department of Anesthesiology and Pain Medicine, Seoul St. Mary’s Hospital, College of Medicine, The Catholic University of Korea, 222, Banpo-daero, Seocho-gu, Seoul 06591, Republic of Korea

**Keywords:** remote ischemic preconditioning, postreperfusion syndrome, epinephrine, living-donor liver transplantation

## Abstract

*Background and Objectives*: Postreperfusion syndrome (PRS) is a significant challenge in liver transplantation (LT), leading to severe circulatory and metabolic complications. Ischemic preconditioning (IPC), including remote IPC (RIPC), can mitigate ischemia-reperfusion injury, although its efficacy in LT remains unclear. This study evaluated the impact of paired RIPC, involving the application of RIPC to both the recipient and the living donor, on the incidence of PRS and the need for rescue epinephrine during living-donor LT (LDLT). *Materials and Methods*: This retrospective observational cohort analysis included 676 adult patients who had undergone elective LDLT between September 2012 and September 2022. After applying exclusion criteria and propensity score matching (PSM), 664 patients were categorized into the paired RIPC and non-RIPC groups. The primary outcomes were the occurrence of PRS and the need for rescue epinephrine during reperfusion. *Results*: The incidence of PRS and the need for rescue epinephrine were significantly lower in the paired RIPC group than in the non-RIPC group. Furthermore, the incidence of postoperative acute kidney injury was lower in the paired RIPC group. Multivariable logistic regression adjusted for propensity scores indicated that paired RIPC was significantly associated with a reduced occurrence of PRS (odds ratio: 0.672, 95% confidence interval: 0.479–0.953, *p* = 0.021). *Conclusions*: Paired RIPC, involving both the recipient and the living donor, effectively reduces the occurrence of PRS and the need for rescue epinephrine during LDLT. These findings suggest that paired RIPC protects against ischemia-reperfusion injury in LDLT. Future randomized controlled trials are needed to verify our results and to explore the underlying mechanisms of the protective effects of RIPC.

## 1. Introduction

Postreperfusion syndrome (PRS) poses a significant challenge in liver transplantation (LT), resulting in serious circulatory and metabolic issues. This condition occurs abruptly when blood flow is restored to the transplanted liver after unclamping the portal vein [1]. Despite advancements in surgical techniques, graft preservation, and anesthesia, the incidence of PRS remains high, affecting approximately 30% of LT patients, with no significant difference between recipients of organs from deceased or living donors [2,3]. The exact causes of PRS are not fully understood. However, severe hemodynamic instability during PRS mainly results from the response of the cardiovascular system to various substances released by the newly grafted liver, including tumor necrosis factor-α, interleukins 1, 2, and 8, which are part of the body’s inflammatory response, and other mediators such as bradykinin, chemokines, and activated complement components that trigger the patient’s immune response upon reperfusion [4]. Furthermore, PRS significantly contributes to the metabolic strain experienced during surgery and anesthesia, often leading to stress-induced hyperglycemia and exacerbating insulin resistance. This condition is evident through intraoperative C-peptide measurements, with blood glucose levels exceeding 200 mg/dL during the neohepatic phase and the first week postoperatively. PRS can further complicate the patient’s recovery and adversely affect the success of the transplantation [5,6].

Ischemic preconditioning (IPC) is a technique designed to protect tissues from ischemia-reperfusion injury by intentionally inducing brief periods of ischemia followed by reperfusion in the same tissue that will later be subjected to a longer ischemic event. IPC was initially identified in heart muscle and has been successfully applied in various tissues, including the liver. IPC involves clamping and unclamping the blood vessels of the target organ to precondition it against subsequent ischemic damage [7,8,9,10]. Remote ischemic preconditioning (RIPC), on the other hand, extends this concept by applying brief ischemia and reperfusion cycles to a tissue or organ remote from the target organ. For instance, in the context of liver transplantation, RIPC can be applied to the limbs (such as the arms or legs) rather than directly to the liver. The protective signals generated during RIPC are thought to be transmitted to the target organ through neural and humoral pathways, thereby conferring protection against ischemia-reperfusion injury [11,12,13,14].

In liver surgery, RIPC is commonly used for warm ischemia during liver resection [8,11,14]. However, its effectiveness in LT has shown mixed results. Despite several clinical trials and a Cochrane review demonstrating the positive impact of IPC on reducing ischemia-reperfusion injury in donor’s liver retrievals, its effects on clinical outcomes remain unclear [12,13,15]. Considering the pathophysiology of PRS, mitigating ischemia-reperfusion injury in liver grafts could potentially benefit patients. However, the application of IPC techniques to liver grafts remains controversial, and no clinical trials have evaluated its effects on the incidence and severity of PRS. Recent animal studies have explored new approaches for IPC, such as RIPC, demonstrating promising results in reducing ischemia-reperfusion injury through intermittent conditioning of tissues other than the target organ [16,17,18]. These advancements may deepen our understanding of ischemia-reperfusion injury and preconditioning mechanisms, serving as a valuable strategy for improving outcomes in LT.

The concept of RIPC has been explored extensively as a method to improve graft quality by reducing ischemia-reperfusion injury. Traditionally, RIPC has been applied solely to the donor, with the aim of preconditioning the donor’s liver to withstand the stresses of transplantation [8,19]. However, recent studies have suggested that a paired approach, involving both the donor and the recipient, might amplify the protective effects of RIPC [9,11,20]. By preconditioning both the donor’s liver and the recipient’s cardiovascular system, this dual strategy aims to enhance the overall resilience against ischemia-reperfusion injury from both ends. The recipient’s preconditioning is hypothesized to better prepare their system for the inflammatory and hemodynamic challenges posed by reperfusion, potentially leading to more stable hemodynamics during this critical phase and reducing the incidence and severity of PRS [10,20,21]. Despite the relatively modest effects observed in some studies, the potential cumulative benefits of paired RIPC warrant further investigation. This study seeks to evaluate the impact of paired RIPC on PRS incidence and the need for rescue epinephrine during living-donor liver transplantation (LDLT), contributing to the growing body of evidence on optimizing both donor and recipient conditions to improve transplantation outcomes.

Therefore, we evaluated the impact of paired RIPC, involving both the recipient and the living donor, on the incidence of PRS and the need for rescue epinephrine during reperfusion in LDLT.

## 2. Patients and Methods

### 2.1. Ethical Considerations

This retrospective observational cohort study was performed under the ethical principles of the Declaration of Helsinki. The study protocol was approved by our Institutional Review Board and Ethics Committee (approval no.: KC21IRSI0576) on 17 September 2021. The study, which adopted a retrospective design necessitating patient enrollment prior to the completion of the registration, was registered on ClinicalTrials.gov (identifier: NCT06312098, Principal investigator: Min Suk Chae, Date of registration: 15 March 2024). Due to the retrospective study design, the requirement for informed consent was waived. The study outcomes are reported in accordance with the Strengthening the Reporting of Observational Studies in Epidemiology Statement.

### 2.2. Study Population

We identified 912 adults aged ≥ 19 years who underwent elective LDLT at our hospital between September 2012 and September 2022. We excluded patients aged < 19 years (pediatric patients) and those with pathological arm findings (such as fracture or skin/subcutaneous injury), arrhythmias (such as atrial fibrillation, ventricular premature complexes, or bundle branch block) in preoperative electrocardiograms, history of percutaneous coronary intervention or use of angina medications (such as aspirin, nitroglycerin, beta-blockers, statins, or calcium channel blockers), deceased-donor or ABO-incompatible kidney transplants, multi-organ transplants including the liver, and re-transplantation. In addition, we excluded individuals with incomplete or missing data related to the recipient or donor graft.

**Figure 1:**
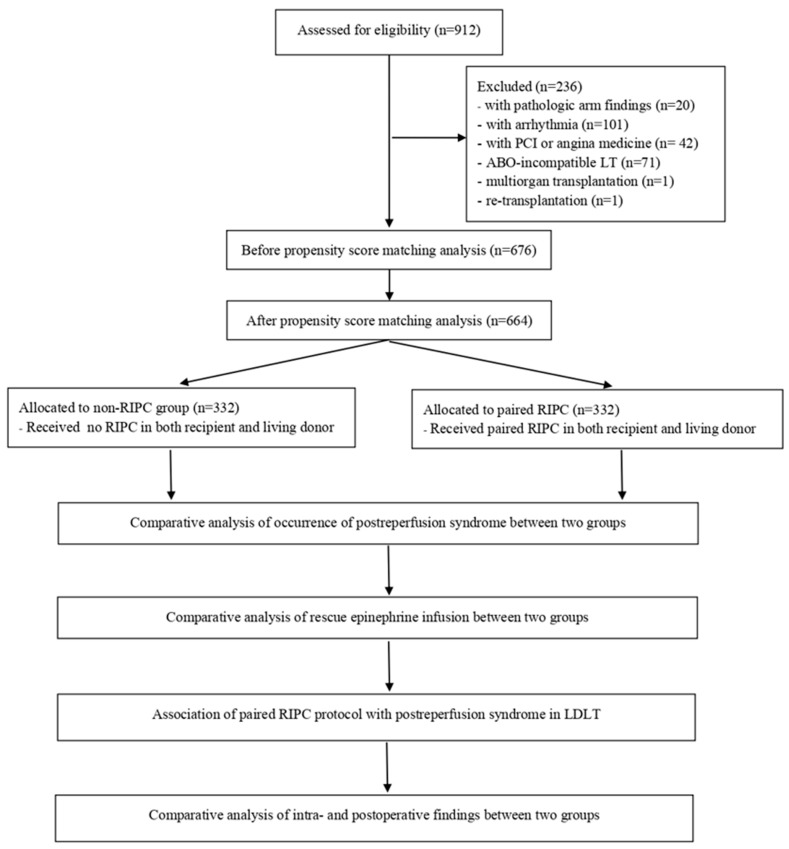
Flow diagram.

Based on the inclusion and exclusion criteria, 236 patients were excluded from the study. Data from the remaining 676 patients were subjected to propensity score matching (PSM), leading to the identification of 664 matched patients. These patients were categorized into two groups based on whether the paired RIPC protocol, involving both recipients and living donors, was employed: the paired RIPC group (*n* = 332) and the non-RIPC group (*n* = 332) (Figure 1).

### 2.3. Surgery and General Anesthesia

The surgical processes and anesthetic care in LDLT have previously been described [22]. In summary, the transplant procedures utilized the piggyback technique, employing the right hepatic lobe along with reconstruction of the middle hepatic vein. The use of venovenous bypass was omitted. Anesthesia during the operations was maintained through a balanced approach, incorporating a volatile agent (desflurane) and opioid medication (fentanyl and remifentanil) for pain control. Muscle relaxation was achieved using neuromuscular blockers (rocuronium) as required.

### 2.4. Paired RIPC in Recipients and Living Donors

Following induction of anesthesia, paired RIPC was simultaneously administered to the upper arms of both the recipient and the living donor. The RIPC procedure utilized a manual cuff inflator to perform three cycles, each consisting of 5 min blood pressure cuff inflation to 250 mmHg or 50 mmHg above the individual’s preoperative systolic blood pressure, whichever was higher [8,19]. These inflation periods were interspersed with 5 min intervals of cuff deflation to allow for recovery. The timing and application of the paired RIPC were carefully coordinated to align with the sequence of the surgical procedures. During this study, we specifically adhered to the paired RIPC protocol (applying RIPC to both the recipient and donor) or omitted RIPC entirely to maintain consistency and evaluate the combined effect of dual RIPC application. No other forms of ischemic preconditioning, such as the intraoperative Pringle maneuver, were performed during donor hepatectomy or liver transplantation procedures.

To ensure consistency and minimize variability, we alternated between applying and not applying RIPC to both the recipient and living donor. All surgeons and anesthesiologists involved in the LT procedures were trained and familiarized with the paired RIPC protocol. This alternating approach and comprehensive training aimed to standardize the application of the procedure across all cases, regardless of the specific personnel involved.

### 2.5. Definition of PRS and Rescue Epinephrine

PRS during LDLT was identified by a significant decrease in mean arterial pressure (MAP), specifically a reduction of ≥30% compared to the MAP at the end of the anhepatic phase, or an absolute MAP < 60 mmHg for ≥1 min within the first 5 min following reperfusion of the transplanted liver. Additionally, an increase in norepinephrine infusion rate by ≥100% within the first 5 min after reperfusion or the need for bolus epinephrine (≥5 μg) for recovery from severe hypotension was also considered indicative of PRS [1].

### 2.6. Clinical Variables

For PSM between the non-RIPC and paired RIPC groups, a comprehensive evaluation of preoperative recipient variables and donor/graft characteristics was conducted. The former factors included age, sex (female), body mass index (BMI), diabetes mellitus, hypertension, cirrhosis-related complications (such as model for end-stage liver disease score, encephalopathy, varix, ascites, and the need for continuous renal replacement therapy), echocardiographic parameters (ejection fraction and diastolic dysfunction), and laboratory parameters (white blood cell count, neutrophil, and lymphocyte counts, hematocrit, levels of aspartate (AST) and alanine (ALT) aminotransferase, total bilirubin, sodium, calcium, potassium, albumin, ammonia, platelet count, and international normalized ratio). The latter variables included donor age, sex (female), BMI, graft-to-recipient weight ratio, graft weight, the extent of change in graft fatty percentage, and graft ischemic time.

Following PSM, the groups were compared based on intra- and postoperative variables. The intraoperative variables were operation duration, average vital signs (including systolic and diastolic blood pressure and heart rate), hourly fluid administration, hourly urine output, and the transfusion of blood products (packed red blood cells, fresh frozen plasma, single donor platelets, and cryoprecipitate). The postoperative variables were the duration of stay in the intensive care unit and total hospital stay and the incidence rates of complications such as acute kidney injury (AKI) [23] and early allograft dysfunction [24]. AKI was defined according to the Kidney Disease: Improving Global Outcomes (KDIGO) criteria. Specifically, AKI was identified as an increase in serum creatinine by ≥0.3 mg/dL (≥26.5 μmol/L) within 48 h, an increase in serum creatinine to ≥1.5 times baseline within the prior 7 days, or urine volume < 0.5 mL/kg/h for 6 h [25].

### 2.7. Statistical Analysis

To assess the distribution of continuous variables, the Shapiro–Wilk test was used, with normally distributed data reported as medians and interquartile ranges (IQRs). Categorical variables are presented as counts and percentages. PSM was employed to reduce the potential influence of confounders, particularly for the paired RIPC group. Propensity scores were calculated to match patients one-to-one using greedy matching algorithms without replacement. Differences in perioperative recipient and donor graft factors between groups were analyzed using the Mann–Whitney *U* test for continuous variables and the *χ*^2^ test or Fisher’s exact test for categorical variables, as appropriate. The impact of paired RIPC on PRS incidence was evaluated through multivariable logistic regression, adjusting for propensity scores. Results are presented as odds ratios (ORs) with 95% confidence intervals (CIs). For survival analysis, Kaplan–Meier survival curves were generated to compare overall and graft survival rates at 3 and 12 months post-LDLT between the paired RIPC and non-RIPC groups. Log-rank tests were used to assess statistical significance between the survival curves. *p*-values < 0.05 were considered indicative of statistical significance. Statistical analyses were performed using R software (version 2.10.1; R Foundation for Statistical Computing, Vienna, Austria) and SPSS for Windows (version 24.0; IBM Corp., Armonk, NY, USA).

## 3. Results

### 3.1. Demographic Characteristics

Of the 676 patients who had undergone LDLT, 28.7% were females. The median age of the patients was 54 (IQR: 48.0–59.8) years, and the median BMI was 24.2 (IQR: 22.0–26.6) kg/m^2^. The leading causes for LDLT were hepatitis B (55.5%), followed by alcoholic hepatitis (20.9%), hepatitis C (7.1%), autoimmune hepatitis (4.4%), hepatitis A (4.3%), drug and toxic hepatitis (1.8%), and cryptogenic hepatitis (6.1%). The median model for end-stage liver disease score was 14.1 (IQR: 6.8–23.7) points.

### 3.2. Comparison of Preoperative Recipient and Donor/Graft Factors Before and After PSM

Before PSM, significant differences were observed between the groups in terms of the preoperative recipient factors, particularly diastolic dysfunction and potassium level (Table 1). However, after PSM, these differences in preoperative characteristics and donor/graft factors were mitigated, demonstrating no significant differences between the groups.

### 3.3. PRS Occurrence and Rescue Epinephrine Requirement

The incidence of PRS was significantly lower in the paired RIPC group compared to the non-RIPC group (27.1% vs. 35.2%, *p* = 0.024) (Table 2). Additionally, the need for rescue epinephrine during reperfusion was significantly reduced in the paired RIPC group (median dose: 20.0 μg vs. 40.0 μg, *p* < 0.001).

Postreperfusion serum potassium levels were measured to assess differences between the groups. The serum potassium levels were not significantly different between the paired RIPC and non-RIPC groups (4.5 ± 0.7 mmol/L vs. 4.6 ± 0.8 mmol/L, *p* = 0.217). Similarly, ALT and AST levels postreperfusion were analyzed. There were no significant differences in ALT levels (45.0 ± 15.2 IU/L vs. 46.5 ± 14.8 IU/L, *p* = 0.371) or AST levels (48.3 ± 16.1 IU/L vs. 49.7 ± 15.5 IU/L, *p* = 0.456) between the paired RIPC and non-RIPC groups.

### 3.4. Association Between Paired RIPC and PRS in PSM Patients

In the propensity score-matched cohort, we conducted a multivariable logistic regression analysis that included the matched propensity score to control for baseline differences. This analysis demonstrated that paired RIPC was significantly associated with a reduced occurrence of PRS, with an odds ratio of 0.672 (95% confidence interval: 0.479–0.953, *p* = 0.021) (Table 3). This finding indicates that the paired RIPC protocol provides a protective effect against the development of PRS during LDLT, even after accounting for potential confounding factors through propensity score adjustment.

### 3.5. Intra- and Postoperative Findings in PS-Matched Patients

The incidence of postoperative AKI, defined according to the Kidney Disease: Improving Global Outcomes (KDIGO) criteria, was also lower in the paired RIPC group (18.1% vs. 25.3%, *p* = 0.024) (Table 4). When separated by KDIGO stages, the incidence of AKI was as follows: KDIGO stage 1: 12.7% (paired RIPC) vs. 18.1% (non-RIPC), *p* = 0.045; KDIGO stage 2: 3.0% (paired RIPC) vs. 5.4% (non-RIPC), *p* = 0.121; KDIGO stage 3: 2.4% (paired RIPC) vs. 1.8% (non-RIPC), *p* = 0.671. Recovery from AKI, defined as the return to baseline serum creatinine levels within 30 days postoperatively, was assessed. The recovery rates were significantly higher in the paired RIPC group compared to the non-RIPC group (83.3% vs. 74.6%, *p* = 0.038). This indicates that not only was the incidence of AKI lower in the paired RIPC group, but the recovery from AKI was also more favorable.

However, there were no significant differences between the two groups in terms of operation time, average systolic and diastolic blood pressures, heart rate, hourly fluid infusion, hourly urine output, and the transfusion of blood products. Postoperative outcomes demonstrated that the duration of stay in the intensive care unit (ICU) and the total hospital stay were not significantly different between the two groups (ICU stay: 7.0 days vs. 6.8 days, *p* = 0.299; hospital stay: 22.0 days vs. 23.0 days, *p* = 0.287). The incidence of early allograft dysfunction was similar between the paired RIPC and non-RIPC groups (14.8% vs. 15.1%, *p* > 0.999).

Overall survival and graft survival were also compared between the two groups at both 3 months and 1 year post-LDLT. The 3-month overall survival rate was 95% in the paired RIPC group and 93% in the non-RIPC group (*p* = 0.67). At 1 year, the overall survival rate was 88.5% in the paired RIPC group and 86.2% in the non-RIPC group (*p* = 0.432), with no significant difference observed. Similarly, the 1-year graft survival rate was 87.1% in the paired RIPC group and 84.9% in the non-RIPC group (*p* = 0.391), indicating no statistically significant differences between the groups in terms of graft survival.

## 4. Discussion

Our findings suggest that paired RIPC can effectively reduce the occurrence of PRS and the need for rescue epinephrine during LDLT. When adjustments were made for propensity scores, the use of paired RIPC was associated with a significant reduction in the PRS rate, with the likelihood of experiencing PRS being 32.8% lower in the paired RIPC group than in the non-RIPC group. In addition, the paired RIPC group also demonstrated a lower incidence of AKI, suggesting an enhanced safety profile of the transplantation surgery.

In a previous randomized clinical trial, Jung et al. [8] investigated the impact of RIPC conducted in living liver donors on postoperative liver function in both donors and recipients. Their findings demonstrated that RIPC in living donors significantly improved postoperative liver function, aligning with our observations on the benefits of paired RIPC in LDLT. Specifically, Jung et al. reported enhanced liver function postoperatively, evidenced by lower AST levels in recipients of preconditioned grafts. Our study builds upon these findings by incorporating a paired RIPC approach, involving both recipients and donors. We observed a significant reduction in the incidence of PRS and the need for rescue epinephrine in the paired RIPC group compared to the non-RIPC group. Additionally, our results indicated a lower incidence of postoperative acute kidney injury in the paired RIPC group, suggesting broader protective effects beyond liver function alone. These findings reinforce the potential clinical benefits of RIPC and support the hypothesis that paired RIPC can enhance overall surgical outcomes in LDLT. While Jung et al.‘s study focused on liver function improvements, our study highlights the additional hemodynamic stability and reduced complication rates associated with paired RIPC. The relatively low model for end-stage liver disease (MELD) scores of recipients in our study cohort might partially explain the observed outcomes. Despite the low MELD scores, the incidences of early allograft dysfunction and AKI highlight the complexity and multifactorial nature of postoperative complications in liver transplantation. Factors such as intraoperative hemodynamic stability, ischemia-reperfusion injury, and preoperative recipient condition likely contribute to these outcomes [26]. Together, these studies underscore the need for further randomized controlled trials to validate the efficacy of RIPC protocols and to explore the underlying mechanisms contributing to their protective effects in LDLT.

The mechanisms through which ischemic conditioning treatments like RIPC mitigate ischemia-reperfusion injury are not fully understood. In LDLT, these treatments may protect the liver through humoral, systemic, and neuronal mechanisms, reducing cell death and inflammation while improving hepatic microcirculation [9]. Previous studies have shown that RIPC in liver donors can enhance post-transplant liver function in recipients without significantly affecting donor outcomes, evidenced by lower AST levels in recipients [8]. Another study found that remote ischemic post-conditioning (RIPostC) significantly reduced postoperative AKI incidence, although it did not improve early postoperative graft function or reduce complications, hospital stay length, or short-term mortality [21]. Our findings align with these studies, indicating improved hemodynamic stability during reperfusion and a lower incidence of AKI, highlighting the potential of RIPC to enhance patient outcomes by reducing intraoperative stress and postoperative complications.

PRS is a significant challenge during LT, impacting both recipient and graft recovery by increasing hemodynamic and metabolic stress [27]. Various strategies have been explored to mitigate PRS. Surgical techniques like the piggyback method maintain better hemodynamic balance and reduce PRS compared to traditional methods such as complete clamping of the inferior vena cava and venovenous bypass [28]. Enhancing hemodynamic stability through methods like flushing the liver graft with lactated Ringer’s solution is also beneficial [29]. Retrograde reperfusion using the piggyback technique lowers PRS incidence and severity, whereas direct reperfusion leads to significant cardiovascular instability [30,31]. Beyond surgical techniques, pharmacological interventions targeting PRS mechanisms have shown promise. Pretreatment with methylene blue maintains the MAP and reduces the need for epinephrine during reperfusion by inhibiting guanylate cyclase [32]. Additionally, pretreatment with low doses of epinephrine or phenylephrine immediately before reperfusion significantly reduces PRS occurrence and the need for vasopressors without adversely impacting the MAP, heart rate, or postoperative recovery compared to control groups [33].

Although strategies to mitigate PRS in LT, such as the piggyback technique and pharmacological interventions, have shown potential, they come with challenges. Surgical methods require extensive training due to a significant learning curve [34], and pharmacological treatments can cause side effects like hypertension or tachycardia, though generally manageable [33]. Balancing the benefits and risks of these approaches necessitates careful monitoring and ongoing research. RIPC, however, is a refined variant of ischemic conditioning with considerable clinical usefulness. Its most significant advantage is providing protective effects without adverse complications. RIPC is a novel, noninvasive strategy for protecting the heart and other organs against ischemia-reperfusion injury, reducing myocardial infarction size, and improving outcomes in acute myocardial infarction and various shock states. It is simple, externally applicable, and useful for early intervention, even before hospital admission. Studies have shown that RIPC activates protective pathways, such as the K(+)-dependent ATP channel, reducing inflammation and enhancing organ function [7]. Since Przyklenk et al. [35] demonstrated its protective effects, numerous clinical trials have explored its therapeutic value. Our research on LDLT suggests that employing paired RIPC, with its simple and convenient approach, can effectively prevent PRS and reduce the need for epinephrine [6]. These findings highlight the potential of RIPC to reduce surgical stress in the recipients, potentially leading to better outcomes and enhanced recovery.

The controversy regarding the effectiveness of RIPC continues, largely due to varying outcomes reported in clinical studies [36,37,38]. This variability may stem from an incomplete understanding of RIPC’s protective mechanisms, differences in study designs, patient comorbidities, types of anesthesia, and surgical techniques [39]. The diversity in patient demographics and numerous potential confounding factors further contribute to inconsistent findings, highlighting the challenges in determining RIPC’s therapeutic value [7]. Despite these conflicting results, RIPC’s simplicity and convenience suggest its clinical usefulness for reducing PRS and the need for epinephrine, thereby correcting hemodynamic instability. These effects are particularly important for improving the recovery outcomes of LDLT patients. Notably, higher doses of epinephrine and lower doses of fentanyl are associated with Takotsubo syndrome, often triggered by intense surgical stress. Our results suggest that RIPC is beneficial for enhancing the recovery of LDLT patients by mitigating these associated risks [40].

In the context of LT, various strategies have been explored to mitigate ischemia-reperfusion injury and improve the outcomes. One such technique is hypothermic machine perfusion, which has been shown to decrease the incidence of PRS by more than 50% in deceased-donor transplants. Hypothermic machine perfusion involves the continuous perfusion of the liver graft with a cold preservation solution, maintaining the organ at low temperatures (typically around 4 °C) to reduce metabolic activity and limit ischemic damage [41,42]. The success of hypothermic machine perfusion in deceased-donor LT raises important questions about its potential application in LDLT. While hypothermic machine perfusion is primarily used to preserve organs during transport and storage in deceased-donor settings, its application in LDLT could theoretically offer similar protective benefits during the period between graft retrieval and implantation. This technique might further reduce ischemia-reperfusion injury and improve graft function, potentially enhancing the outcomes observed with paired RIPC. However, several challenges must be addressed before incorporating hypothermic machine perfusion into LDLT. These include the logistical complexities of setting up and maintaining perfusion systems in a living-donor setting, as well as the need for clinical trials to evaluate the safety and efficacy of this approach in LDLT. Additionally, ethical considerations surrounding the use of invasive perfusion techniques in living donors must be carefully weighed. Despite these challenges, the potential benefits of hypothermic machine perfusion in reducing PRS and improving graft outcomes warrant further investigation. Future research should explore the feasibility and impact of combining hypothermic machine perfusion with paired RIPC in LDLT, potentially offering a synergistic approach to optimize graft preservation and recipient outcomes.

Our study has several limitations that should be considered when interpreting the results. First, as a retrospective observational cohort study, there is an inherent risk of selection and indication bias. Although we employed PSM to minimize the impact of confounding variables, the decision to implement paired RIPC was not randomized and may have been influenced by evolving institutional protocols and clinical judgment over the study period. This could introduce indication bias, as the choice to use paired RIPC may have been influenced by patient characteristics or intraoperative factors not fully accounted for in the PSM process. Second, the study’s retrospective design limits our ability to establish causality. While our findings suggest a protective effect of paired RIPC on PRS and AKI, prospective randomized controlled trials are needed to confirm these results and better understand the underlying mechanisms. Third, our study focused exclusively on LDLT and may not be generalizable to deceased-donor LT or other types of organ transplants. The specific protocols and outcomes associated with paired RIPC in LDLT may differ from those in other transplant settings. Finally, the follow-up period was limited to 1 year, which may not capture long-term outcomes and complications associated with paired RIPC. Future studies with longer follow-up durations are necessary to evaluate the sustained impact of paired RIPC on graft and patient survival. Despite these limitations, our study provides valuable insights into the potential benefits of paired RIPC in reducing PRS and improving postoperative outcomes in LDLT. Further research, particularly randomized controlled trials, is essential to validate our findings and refine the application of RIPC protocols in LT.

## 5. Conclusions

Our investigation into paired RIPC has demonstrated its potential to significantly reduce PRS and the need for epinephrine in LDLT. While promising, our results highlight the need for further research to understand the underlying mechanisms of RIPC and to establish standardized clinical guidelines. Our study had limitations, including its narrow focus on LDLT and potential confounding factors, which may affect the generalizability of our findings. Well-designed randomized controlled trials are needed to confirm the therapeutic benefits of RIPC across all types of liver transplantation surgeries and to understand its impact on PRS risk factors. Despite these limitations, our results suggest that RIPC enhances surgical outcomes in LT. Based on the positive findings of this study, paired RIPC has been incorporated into the standard practice at our institution. We now regularly perform paired RIPC for all eligible living-donor liver transplantations, reflecting our commitment to improving patient outcomes through evidence-based practices.

## Figures and Tables

**Table 1:** Comparison of demographic factors between the non-RIPC and paired RIPC groups before and after PSM.

	Before Propensity Score Matching				After Propensity Score Matching			
Group	Non-RIPC	Paired RIPC	*p*	SD	Non-RIPC	Paired RIPC	*p*	SD
***n***	**338**	**338**			**332**	**332**		
**Recipient variables**								
Age (years)	53.0 (47.0–60.0)	54.0 (48.0–59.3)	0.412	0.053	53.0 (47.0–60.0)	54.0 (48.0–59.0)	0.505	0.040
Sex (female)	98 (29.0%)	96 (28.4%)	0.865	−0.013	97 (29.2%)	93 (28.0%)	0.731	−0.027
Body mass index (kg/m^2^)	24.2 (22.0–26.6)	24.2 (22.0–26.6)	0.984	0.004	24.2 (22.0–26.6)	24.2 (22.1–26.6)	0.978	−0.003
Comorbidities								
Hypertension	69 (20.4%)	68 (20.1%)	0.924	−0.007	67 (20.2%)	67 (20.2%)	>0.999	0.000
Diabetes mellitus	83 (24.6%)	98 (29.0%)	0.193	0.098	83 (25.0%)	96 (28.9%)	0.256	0.086
Cirrhotic complications								
MELD score (points)	14.5 (6.5–24.5)	13.6 (7.1–23.0)	0.938	−0.023	14.4 (6.6–24.2)	13.6 (7.1–23.0)	0.825	−0.009
Encephalopathy (West Haven criteria I or II)	119 (35.2%)	130 (38.5%)	0.38	0.067	117 (35.2%)	129 (38.9%)	0.335	0.074
Varix	80 (23.7%)	88 (26.0%)	0.476	0.054	79 (23.8%)	86 (25.9%)	0.53	0.048
Ascites ≥ 1 L	156 (46.2%)	165 (48.8%)	0.488	0.053	154 (46.4%)	162 (48.8%)	0.534	0.048
Continuous renal replacement therapy	54 (16.0%)	41 (12.1%)	0.15	−0.118	53 (16.0%)	41 (12.3%)	0.182	−0.111
Echocardiographic findings								
Ejection fraction (%)	64.4 (62.0–66.4)	64.7 (62.0–67.0)	0.023	0.127	64.4 (62.0–66.5)	64.4 (62.0–67.0)	0.086	0.135
Diastolic dysfunction (≥grade II)	53 (15.7%)	56 (16.6%)	0.754	0.024	53 (16.0%)	54 (16.3%)	0.916	0.008
Laboratory findings								
White blood cell count (×10^9^/L)	4.5 (2.9–7.2)	4.5 (2.9–7.6)	0.771	0.028	4.5 (2.9–7.2)	4.5 (2.9–7.5)	0.785	−0.008
Neutrophil (%)	61.8 (52.7–74.7)	60.9 (50.5–72.8)	0.098	−0.150	61.6 (52.3–74.6)	60.7 (50.5–72.9)	0.143	−0.136
Lymphocyte (%)	21.0 (11.8–31.2)	23.2 (13.1–33.4)	0.132	0.129	21.2 (11.8–31.6)	23.2 (13.0–33.5)	0.183	0.117
Hematocrit (%)	29.4 (24.6–35.6)	29.5 (25.4–34.9)	0.872	0.004	29.4 (24.5–35.5)	29.5 (25.4–34.9)	0.839	0.007
Aspartate aminotransferase (IU/L)	46.0 (32.8–85.0)	48.0 (33.0–86.8)	0.783	−0.024	46.0 (32.0–85.0)	48.0 (33.0–89.8)	0.681	−0.024
Alanine aminotransferase (IU/L)	32.0 (21.0–59.0)	30.0 (21.0–58.0)	0.587	−0.022	32.5 (21.0–59.8)	30.0 (21.0–57.8)	0.59	−0.022
Total bilirubin (mg/dL)	2.4 (0.9–14.1)	2.5 (1.0–12.7)	0.405	−0.044	2.3 (0.9–13.5)	2.5 (1.0–12.8)	0.299	−0.021
Sodium (mEq/L)	139.0 (135.0–142.0)	139.0 (135.0–141.0)	0.129	−0.119	139.0 (135.0–142.0)	139.0 (135.0–141.0)	0.113	−0.124
Calcium (mg/dL)	8.4 (8.0–8.8)	8.3 (7.8–8.8)	0.116	−0.065	8.4 (8.0–8.8)	8.3 (7.8–8.8)	0.144	−0.058
Potassium (mEq/L)	3.9 (3.6–4.3)	4.0 (3.7–4.4)	0.024	0.153	3.9 (3.6–4.3)	4.0 (3.7–4.4)	0.087	0.147
Albumin (g/dL)	3.1 (2.7–3.6)	3.0 (2.7–3.5)	0.119	−0.100	3.1 (2.7–3.6)	3.0 (2.7–3.5)	0.153	−0.093
Ammonia (ug/dL)	95.0 (64.0–143.3)	100.5 (68.0–162.0)	0.104	0.124	96.0 (65.0–143.8)	100.0 (68.0–162.0)	0.153	0.116
Platelet count (×10^9^/L)	65.0 (46.0–109.0)	64.5 (45.8–99.3)	0.287	−0.240	64.0 (46.0–105.0)	65.0 (46.0–98.8)	0.505	−0.170
International normalized ratio	1.5 (1.2–2.1)	1.5 (1.3–2.1)	0.354	0.043	1.5 (1.2–2.1)	1.5 (1.3–2.1)	0.341	0.046
**Donor variables**								
Age (years)	35.4 (26.8–43.0)	35.2 (26.0–41.0)	0.605	−0.054	35.4 (26.3–43.0)	35.0 (26.0–41.0)	0.595	−0.059
Sex (female)	112 (33.1%)	107 (31.7%)	0.681	−0.032	110 (33.1%)	104 (31.3%)	0.618	−0.039
Body mass index (kg/m^2^)	20.2 (18.4–21.2)	20.2 (18.3–21.6)	0.462	0.055	20.2 (18.3–21.2)	20.2 (18.3–21.6)	0.467	0.046
Graft–recipient weight ratio (%)	1.2 (1.0–1.5)	1.2 (1.0–1.6)	0.694	−0.018	1.2 (1.0–1.5)	1.2 (1.0–1.6)	0.792	−0.012
Graft weight (g)	834.0 (691.5–936.5)	821.0 (701.5–980.0)	0.877	−0.025	834.0 (690.5–935.5)	820.0 (700.5–979.0)	0.912	−0.022
Graft fatty change (%)	4.9 (1.0–5.0)	4.9 (1.0–5.0)	0.045	0.100	4.9 (1.0–5.0)	4.9 (1.0–5.0)	0.062	0.072
Graft ischemic time (min)	93.0 (70.0–128.0)	94.0 (68.0–128.3)	0.748	−0.138	92.5 (69.3–127.8)	94.0 (68.0–127.8)	0.884	−0.099

Abbreviations: PSM, propensity score matching; RIPC, remote ischemic conditioning; SD, standard deviation; MELD, model for end-stage liver disease. Values are expressed as numbers (percentages) and median (interquartile).

**Table 2:** Comparison of PRS-related outcomes between the non-RIPC and paired RIPC groups in propensity score-matched patients.

Group	Non-RIPC	Paired RIPC	*p*
	**332**	**332**	
Incidence of postreperfusion syndrome	117 (35.2%)	90 (27.1%)	0.024
*In patients with PRS (n = 207)*			
Rescue epinephrine infusion (mcg)	40.0 (20.0–50.0)	20.0 (20.0–30.0)	<0.001

Abbreviations: RIPC, remote ischemic preconditioning; PRS, postreperfusion syndrome. Values are expressed as numbers (percentages) and median (interquartile).

**Table 3:** Association between paired RIPC and PRS in LDLT patients.

	*β*	Odds Ratio	95% CI	*p*
***Paired RIPC adjusted for PS***				
Postreperfusion syndrome	−0.398	0.672	0.479–0.953	0.021

Abbreviations: CI, confidence interval; RIPC, remote ischemic preconditioning; PS, propensity score; PRS, postreperfusion syndrome; LDLT, living-donor liver transplantation.

**Table 4:** Comparison of intra- and postoperative findings between the non-RIPC and paired RIPC groups in propensity score-matched patients.

Group	Non-RIPC	Paired RIPC	*p*
	**332**	**332**	
***Intraoperative finding***			
Operation time (min)	500.0 (440.0–570.0)	490.0 (445.0–555.0)	0.325
Average of vital signs			
Systolic blood pressure (mmHg)	108.0 (98.5–115.8)	106.0 (97.5–116.3)	0.424
Diastolic blood pressure (mmHg)	57.8 (52.3–63.3)	56.7 (51.0–62.3)	0.076
Heart rate (beats/min)	88.5 (79.5–97.3)	88.8 (79.8–100.0)	0.819
Hourly fluid infusion (mL/kg/h)	10.8 (8.1–14.6)	10.8 (8.2–14.9)	0.986
Hourly urine output (mL/kg/h)	1.2 (0.6–2.0)	1.3 (0.6–2.2)	0.462
Blood products transfusion (unit)			
Packed red blood cells	8.0 (4.0–14.8)	8.0 (4.0–12.0)	0.395
Fresh frozen plasma	8.0 (4.0–11.0)	7.0 (5.0–10.0)	0.662
Single donor platelet	0.0 (0.0–5.0)	0.0 (0.0–5.0)	0.485
Cryoprecipitate	0.0 (0.0–0.0)	0.0 (0.0–0.0)	0.109
***Postoperative findings***			
ICU stay (day)	6.8 (5.0–7.0)	7.0 (5.0–7.0)	0.299
Hospital stay (day)	23.0 (21.0–30.0)	22.0 (21.0–28.5)	0.287
Incidence of acute kidney injury	84 (25.3%)	60 (18.1%)	0.024
Incidence of early allograft dysfunction	50 (15.1%)	49 (14.8%)	>0.999

Abbreviations: RIPC, remote ischemic preconditioning; ICU, intensive care unit. Values are expressed as numbers (percentages) and median (interquartile).

## Data Availability

Data is contained within the article or Supplementary Materials.

## Supplementary Materials

The following supporting information can be downloaded at: https://www.mdpi.com/article/10.3390/medicina60111830/s1, File S1: STROBE statement.

## Abbreviations

RIPC; remote ischemic preconditioning; LDLT; living-donor liver transplantation; PRS; postreperfusion syndrome; PSM; propensity score matching; PS; propensity score; BMI; body mass index; DM; diabetes mellitus; HTN; hypertension; MELD; model for end-stage liver disease.
